# Chaperone-Usher Fimbriae of *Escherichia coli*

**DOI:** 10.1371/journal.pone.0052835

**Published:** 2013-01-30

**Authors:** Daniël J. Wurpel, Scott A. Beatson, Makrina Totsika, Nicola K. Petty, Mark A. Schembri

**Affiliations:** Australian Infectious Diseases Research Centre, School of Chemistry and Molecular Biosciences, The University of Queensland, Brisbane, Queensland, Australia; Pasteur Institute, France

## Abstract

Chaperone-usher (CU) fimbriae are adhesive surface organelles common to many Gram-negative bacteria. *Escherichia coli* genomes contain a large variety of characterised and putative CU fimbrial operons, however, the classification and annotation of individual loci remains problematic. Here we describe a classification model based on usher phylogeny and genomic locus position to categorise the CU fimbrial types of *E. coli*. Using the BLASTp algorithm, an iterative usher protein search was performed to identify CU fimbrial operons from 35 *E. coli* (and one *Escherichia fergusonnii*) genomes representing different pathogenic and phylogenic lineages, as well as 132 *Escherichia spp*. plasmids. A total of 458 CU fimbrial operons were identified, which represent 38 distinct fimbrial types based on genomic locus position and usher phylogeny. The majority of fimbrial operon types occupied a specific locus position on the *E. coli* chromosome; exceptions were associated with mobile genetic elements. A group of core-associated *E. coli* CU fimbriae were defined and include the Type 1, Yad, Yeh, Yfc, Mat, F9 and Ybg fimbriae. These genes were present as intact or disrupted operons at the same genetic locus in almost all genomes examined. Evaluation of the distribution and prevalence of CU fimbrial types among different pathogenic and phylogenic groups provides an overview of group specific fimbrial profiles and insight into the ancestry and evolution of CU fimbriae in *E. coli*.

## Introduction

Fimbriae are long proteinaceous organelles that extend from the surface of many bacteria and mediate diverse functions, including adherence and biofilm formation. Fimbrial adhesins, which are often located at the tip of the organelle, typically recognize specific receptor targets in a lock-and-key fashion, thus enabling the bacterium to target a specific surface and display tissue tropism. Many different types of fimbriae have been described in Gram-positive and Gram-negative bacteria [1]. In Gram-negative bacteria, fimbriae are assembled via a range of different protein translocation systems, including the chaperone-usher (CU) pathway, the type IV secretion pathway and the extracellular nucleation precipitation pathway [2].

Among the fimbrial types produced by Gram-negative bacteria, the CU class of fimbriae is the most abundant. The genes encoding for CU fimbriae are found in most members of the *Enterobacteriaceae* (e.g. *Escherichia coli*, *Salmonella* spp., *Klebsiella* spp., *Proteus* spp., *Enterobacter* spp., *Citrobacter* spp.) as well as bacteria from other genera including *Pseudomonas, Haemophilus, Bordetella, Burkholderia* and *Acinetobacter* [3], [4]. The CU pathway is a highly conserved bacterial secretion system for the assembly of fimbriae on the bacterial cell surface. Fimbrial biogenesis by the CU pathway requires a periplasmic chaperone and an outer membrane assembly platform termed the usher. The chaperone facilitates several essential steps in the pathway; it mediates the folding of fimbrial subunit proteins, prevents their polymerization in the periplasm and directs their passage to the usher. The usher in turn acts as an assembly platform; it forms a binding scaffold for fimbrial subunit protein-chaperone complexes from the periplasm and facilitates the assembly of the fimbrial structural organelle [5], [6], [7], [8], [9], [10], [11].

The prototypical CU fimbriae are type 1 and P fimbriae from uropathogenic *Escherichia coli* (UPEC), which mediate binding to specific receptors in the bladder and upper urinary tract, respectively, via an adhesin located at the tip of the organelle. The biogenesis, regulation and function of type 1 and P fimbriae have been comprehensively studied [12], [13], [14], [15], [16], [17]. Type 1 fimbriae are 0.2–2.0 μm long tubular structures predominantly comprised of a major structural subunit (FimA) and containing a tip fibrillum composed of several minor components including the FimH adhesin [16], [18], [19]. Type 1 fimbriae confer binding to α-D-mannosylated proteins such as uroplakins, which are abundant in the bladder [20]. The expression of type 1 fimbriae by UPEC enhances colonization and host response induction in the murine urinary tract infection (UTI) model, and promotes biofilm formation and host cell invasion [21], [22], [23]. Like type 1 fimbriae, P fimbriae are composed of a major structural protein (PapA), however they contain a larger tip fibrillum, which is comprised of major (PapE) and minor (PapF, PapK, PapG) components. P fimbriae are strongly associated with acute pyelonephritis; they contribute to the establishment of UTI by binding to the α-D-galactopyranosyl-(1–4)-β-D-galactopyranoside receptor epitope in the globoseries of glycolipids and activate innate immune responses in animal models and in human infection [24], [25], [26], [27].

*E. coli* represents the most comprehensively studied organism with respect to CU fimbriae. In addition to type 1 and P fimbriae, many other CU fimbriae have been characterised and often the adherence properties of these fimbriae are associated with certain *E. coli* pathotypes. For example, P, F1C and S fimbriae are commonly associated with extra-intestinal *E. coli* (ExPEC; including UPEC and meningitis-associated *E. coli* [NMEC]) [26], [28], [29], aggregative adherence fimbriae (AAF) are associated with enteroaggregative *E. coli* (EAEC) [30], long polar fimbriae (LPF) with enteropathogenic *E. coli* (EPEC) and enterohaemorrhagic *E. coli* (EHEC) [31], CS1-CFA/I are associated with human enterotoxigenic *E. coli* [32] and K88 (F4) and K99 (F5) fimbriae with porcine, bovine and ovine enterotoxigenic *E. coli* (ETEC) [33], [34]. The significant increase in bacterial genome sequencing that has occurred over the last decade has also resulted in the identification of many CU fimbrial gene clusters that remain uncharacterised. This includes CU fimbriae from commensal *E. coli* strains, where the expression of many CU fimbriae is cryptic and repressed by the histone-like protein H-NS [35].

Early attempts to distinguish between different types of fimbriae from *E. coli* and other Gram-negative bacteria were based either on morphology, function or serology [36], [37], [38]. More recently, a phylogenetic clade system was established that defines CU fimbriae according to evolutionary descent [3]. In this scheme, CU fimbriae phylogeny is based on the sequence of the usher protein due to its ubiquitous association with all CU gene clusters and the fact that the usher-encoding gene is present in a single copy in all CU gene loci. Here we have employed the classification scheme developed by Nuccio *et* *al*. [3] to define the repertoire of CU fimbriae in *E. coli*. Thirty five *E. coli* (and one *E. fergusonnii*) genomes representing commensal, diarrheagenic and ExPEC strains were searched for genes encoding putative fimbrial usher proteins. A total of 458 usher-encoding genes were identified and individually interrogated for the presence of an adjacent cognate chaperone-encoding gene as well as at least one fimbrial subunit-encoding gene. The CU fimbrial genes were analysed for their distribution, genetic conservation and genetic location among *E. coli* pathotypes.

## Methods

### Identification of Chaperone-Usher Operons

The NCBI BLAST2.2.25+ program [39] was utilised to examine two datasets, one consisting of the whole genomes (chromosomes and plasmids) of 36 *Escherichia* strains (Table 1) and the second dataset containing 132 *Escherichia* plasmids (with no associated chromosome sequence available) (Table S1), for the presence of usher sequences. All amino acid sequences encoded by the genomes and plasmids listed in Table 1 and S1 were downloaded from UniProt [40] and used to build a local BLAST database. The 10 usher amino acid sequences annotated in *E. coli* CFT073 [41], [42] were used as an initial BLASTp query dataset to probe the local BLAST database. BLASTp searches were performed using the BLOSUM62 series algorithm and an E-value cut-off score of 0.1. Newly identified proteins with a reported E-value of 0 were directly added to the usher database, hits with an E-value >0 were screened for the presence of an usher protein family domain (PF00577) and/or flanking chaperone (PF00345, PF02753 or COG3121) encoding genes before they were added to the usher query dataset. The NCBI Conserved Domain Database (CDD) was used to examine amino acid sequences for conserved domains [43]. After each BLASTp run, the updated usher query dataset was used to re-probe the genome and plasmid sequences until no new usher sequences were found.

**Table 1:** *Escherichia* genomes analysed and CU fimbrial operons identified per strain.

*E.coli*	Phylogroup	Total CU Operons	Intact CU Operons	Reference
**UPEC**				
CFT073	B2	12	11	Welch *et al*. 2002 [41]
536	B2	14	12	Hochhut *et al*. 2006 [70]
F11	B2	13	11	Rasko *et al*. 2008 [71]
UTI89	B2	12	10	Chen *et al*. 2006 [72]
EC958	B2	10	9	Totsika *et al*. 2011 [73]
UMN026	D	12	12	Touchon *et al*. 2009 [62]
IAI39	D	14	11	Touchon *et al*. 2009 [62]
**ABU**				
83972	B2	12	9	Zdziarski *et al*. 2010 [74]
**NMEC**				
S88	B2	10	8	Touchon *et al*. 2009 [62]
**APEC**				
APEC01	B2	10	9	Johnson *et al*. 2006 [75]
**EAEC**				
55989	B1	16	14	Touchon *et al*. 2009 [62]
042	D	12	11	Chaudhuri *et al*. 2010 [76]
**EPEC**				
O127:H6 E2348/69	B2	9	8	Iguchi *et al*. 2009 [77]
O55:H7 CB9615	E	14	12	Zhou *et al*. 2010 [78]
**ETEC**				
O78:H11 H10407	A	13	8	Crossman *et al*. 2010 [79]
E24377A	B1	15	12	Rasko *et al*. 2008 [71]
**EHEC**				
O26:H11 11368	B1	15	14	Ogura *et al*. 2009 [80]
O103:H2 12009	B1	14	10	Ogura *et al*. 2009 [80]
O111:H-11128	B1	14	12	Ogura *et al*. 2009 [80]
O157:H7 EDL933	E	14	12	Perna *et al*. 2001 [81]
O157:H7 Sakai	E	14	12	Hayashi *et al*. 2001 [82]
O157:H7 EC4115	E	14	11	Eppinger *et al*. 2011 [83]
O157:H7 TW14359	E	14	11	Kulasekara *et al*. 2009 [84]
**Commensal**				
ATCC 8739	A	11	9	Joint Genome Institute [85]
IAI1	B1	15	14	Touchon *et al*. 2009 [62]
ED1a	B2	10	5	Touchon *et al*. 2009 [62]
HS	A	13	9	Rasko *et al*. 2008 [71]
SE11	B1	17	16	Oshima *et al*. 2008 [86]
SE15	B2	9	8	Toh *et al*. 2010 [87]
**Environmental**				
SMS-3-5	D	12	10	Fricke *et al*. 2008 [88]
**Laboratory**				
BL21(DE3)	A	12	9	Jeong *et al*. 2009 [89]
B REL606	A	12	9	Jeong *et al*. 2009 [89]
K-12 MG1655	A	12	9	Blattner *et al*. 1997 [45]
K-12 DH10β	A	11	8	Durfee *et al*. 2008 [90]
K-12 BW2952	A	11	8	Ferenci *et al*. 2009 [91]

*E.fergusonnii*		Total CU Operons	Intact CU Operons	Reference
**Commensal**				
EFER ATCC 35469	-	7	7	Touchon *et al*. 2009 [62]

UPEC: uropathogenic *E.coli*, ABU: asymptomatic bacteriuria *E.coli*, NMEC: neonatal meningitis *E.coli*, APEC: avian pathogenic *E.coli*, EAEC: enteroaggregative *E.coli*, EPEC: enteropathogenic *E.coli*, EHEC: enterohaemorrhagic *E.coli*.

### Operon Structure Prediction and Analysis of Genetic Context

To determine the genetic organisation of an operon, flanking regions of usher nucleotide sequences were visualised in xBASE [44]. Fimbrial encoding genes were identified using conserved protein domain searches [43] and sequence homology to annotated genes. Intergenic regions >200 bp were investigated for the presence of protein encoding sequences with conserved fimbrial domains or significant sequence identity to fimbrial subunits. To determine the locus position of chromosome-borne fimbrial operons, the genetic context of each operon was visualised in xBASE and aligned with the genome of *E. coli* K-12 MG1655 [45]. Plasmid-borne fimbrial operons were compared to the closest homologous annotated fimbrial sequences, and analysed for genetic organisation and subunit sequence similarity.

### Multiple Sequence Alignment and Phylogenetics

Full-length usher amino acid sequences from intact fimbrial operons (as well as the Yhc and AAF/II ushers) were used to infer evolutionary relationships. Sequences were aligned in ClustalX2.1 [46] using BLOSUM30 for pair-wise alignment with a gap opening penalty of 10 and gap extension penalty of 0.1, and the BLOSUM series matrix for multiple alignment with a gap opening penalty of 10 and a gap extension penalty of 0.2 (default parameters). Phylogenetic analyses were performed with the MEGA5 software package [47]. Protein distance matrices were predicted using the Poisson correction model with default settings. The Neighbour-Joining method was used to generate a phylogenetic tree, which was displayed as an unrooted phylogram using iTOL [48]. To estimate the confidence in the tree topology, a bootstrap test of 1000 replicates was performed. Alignment and phylogenetic tree construction was repeated with usher sequences of previously published usher phylograms [3] to verify tree validity (data not shown).

The evolutionary relationship of the 35 *E. coli* and one *E. fergusonnii* strains included in our analysis was predicted by Multi-Locus Sequence Typing (MLST) of the concatenated nucleotide sequences of 7 housekeeping genes (*adk*, *fumC*, *gyrB*, *icd*, *mdh*, *purA*, *recA*) as previously described [49]. MLST data of *Salmonella enterica* serovar Typhimurium LT2 [50] was incorporated as representative for the *Salmonella* outgroup. Sequences were aligned in ClustalX2.1 using the ClustalW(1.6) DNA weight matrix under default settings. The Neighbour-Joining method of MEGA5 was used to infer the evolutionary history, with distances computed by the Jukes-Cantor method. The resulting phylogenetic tree was visualised in iTOL [48] as a rooted phylogram.

## Results and Discussion

### Identification of CU fimbrial operons in *Escherichia*

A bioinformatic approach was used to identify CU fimbrial gene clusters in *Escherichia*. Fimbrial operons were identified using an iterative usher BLASTp search against a selection of 36 *Escherichia* complete genomes and 132 *Escherichia* spp. plasmids (Table 1 and S1). These genomes represent strains from ExPEC, diarrheagenic and commensal *E. coli*, as well as *Escherichia fergusonnii*. Fimbrial operons were defined as polycistronic gene clusters containing at least an usher and a chaperone encoding sequence, and flanked by one or more genes encoding fimbrial subunits. Usher genes of disrupted operons may be subject to increased change, potentially distorting our interpretation of the phylogenetic relationships amongst usher proteins [51]. To prevent potential bias, CU operons that contained transposon insertion elements or truncated structural genes were considered disrupted and excluded from the evolutionary phylogeny analysis.

A total of 458 CU fimbrial gene clusters were identified from the combined whole genome and plasmid-only datasets. In the whole genome dataset, 449 operons containing usher and chaperone encoding sequences were identified (average 12±2.14 operons per strain; maximum 17, minimum 7) (Table 1 and Table S2). Analysis of the genetic organisation of these CU fimbrial gene clusters revealed that 370 operons were intact (average 10±2.28 intact operons per strain; maximum 16, minimum 5). The vast majority of fimbrial gene clusters in the whole genome dataset were chromosomally located (442/449), while 7 CU fimbrial gene clusters were located on plasmids. In the plasmid-only dataset, another nine CU fimbrial gene clusters were identified, all of which appeared to be intact (Table S2). No orphan usher encoding genes were discovered.

### Classification of *Escherichia* fimbriae

**Figure 1:**
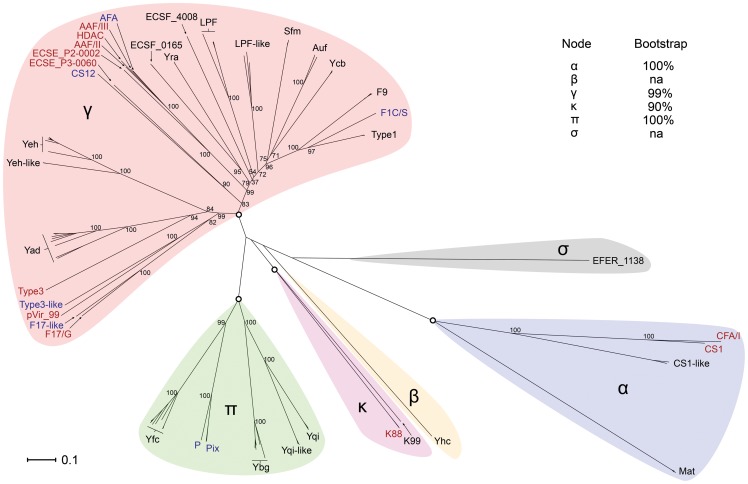
Unrooted phylogram of fimbrial usher proteins identified in *Escherichia*. A total of 1075 amino acid positions were used to infer the evolutionary relationship of 383 aligned usher proteins. These consist of 379 usher amino acid sequences belonging to intact fimbrial gene clusters and an additional four usher amino acid sequences of disrupted fimbrial gene clusters (Yhc and AAF/II), which lack intact representatives in the genome sequenced strains examined in this dataset. The corresponding 383 fimbrial gene clusters can be classified as 38 types based on the evolutionary phylogeny of usher amino acid sequence and genetic locus position. Fimbrial gene clusters were grouped according to the Nuccio clade system (α, β, γ, π, κ, σ, open circles represent cladistic nodes) [3], and highlighted in colour. The text of fimbrial types located on PAI's or plasmids is highlighted in blue and red, respectively. Bootstrap values (1000) are displayed as percentage on major nodes. The scale represents the number of amino acid substitutions per site.

To display the evolutionary relationship of the CU fimbriae usher amino acid sequences, an unrooted phylogram was constructed (Figure 1). This analysis included all the 379 usher amino acid sequences from the intact operons described above, as well as four usher sequences from disrupted operons that lacked intact representatives in the dataset (i.e. Yhc, and AAF/II). The CU clading scheme described previously by Nuccio et al. divides Gram-negative CU fimbriae into six clades (α, β, γ, κ, π, σ) and five sub-clades (γ1, γ2, γ3, γ4 and γ*), based on the evolutionary phylogeny of usher protein sequences [3]. A phylogenetic tree of *Escherichia* usher sequences based on the Nuccio scheme demonstrated that the *Escherichia* genus contains representatives of all six clades, which were labelled accordingly (Figure 1). The γ clade was the largest and encompassed 24 CU fimbrial types across five sub-clades, with the best-characterised fimbriae represented by type 1 fimbriae. The π clade contained 6 CU fimbriae, including the well-characterised P fimbriae from UPEC. The remaining four clades (α, β, κ, σ) comprised relatively few CU fimbrial types. The α clade was the most distantly related, and this is consistent with the classification of CS1-CFA/I fimbriae as members of an alternate CU pathway [52].

**Figure 2:**
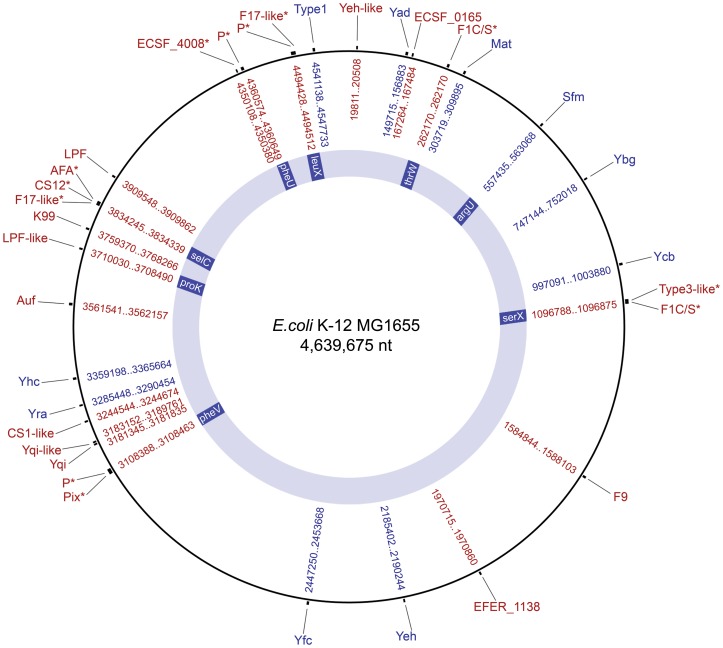
Locus positions of chromosome-borne CU fimbrial operons relative to the genome of *E.coli* MG1655. The *E. coli* K-12 MG1655 chromosome (outer black ring) was used as a reference map to visualise the locus position of 30 chromosome-borne CU fimbrial types. Types highlighted in blue are present in *E. coli* K-12 MG1655, types in red are absent in this strain. Fimbrial types associated with PAIs are indicated by an asterisk. A number of PAI associated fimbrial gene clusters occupy different locus positions relative to the MG1655 genome. tRNA sites that flank CU-containing PAIs are indicated on the inner blue ring.

The majority of CU fimbrial operons showed a strong relationship between chromosomal location and usher phylogeny. Accordingly, we superimposed the operon locus of each chromosomal CU type on the *E. coli* MG1655 reference genome (Figure 2). Based on usher phylogeny and locus position, the 458 CU operons identified in *Escherichia* can be classified as 38 fimbrial types. CU fimbrial genes that could not be mapped in this manner were either located on plasmids (i.e. CS1-CFA/I, ECSE_P2-002, ECSE_P3-0031, K88, AAF) or within pathogenicity-associated islands (PAIs) (i.e. P, F1C, S, Pix and F17-like fimbriae), which are known to exist at various insertion sites on the *E. coli* chromosome backbone. CU operons associated with these mobile elements were typed according to usher phylogeny and conservation of their genetic organisation.

### Genetic organisation of CU fimbrial gene clusters

**Figure 3:**
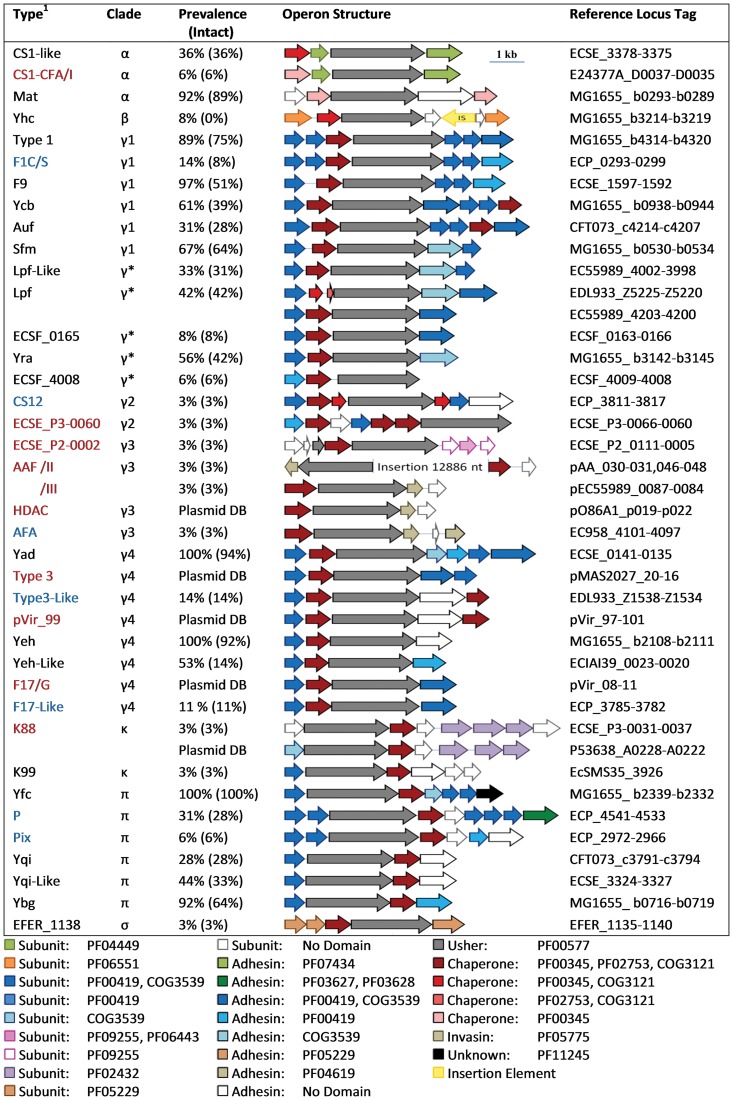
Prevalence and genetic organisation of CU fimbrial types identified in *Escherichia*. The genetic organisation of the different fimbrial types is depicted diagrammatically. Fimbriae are grouped according to the Nuccio clading scheme [3]. Fimbrial prevalence is represented as a percentage of all the strains in the genome dataset. Plasmid-borne fimbriae not part of a genome are highlighted as ‘Plasmid DB’. Genes are colour coded according to predicted function of the corresponding protein product, with associated Pfam and COG domains indicated. The scale represents DNA length in kilo base pair. Reference operon locus tags for individual fimbrial types are displayed on the right. ^1^PAI and plasmid-borne operons are highlighted in blue and red, respectively.

The genetic organisation of the CU fimbrial gene clusters was predicted by reviewing the literature and inspecting individual genes for conserved fimbrial protein domains (Figure 3). In most instances, the genetic structure of operons belonging to the same fimbrial type was conserved. The exceptions were Lpf and K88 fimbriae, where additional subunit genes have been acquired or lost in certain strains. For example, in EHEC O157:H7 and EPEC O55:H7 strains the *lpf* operon contains an additional gene encoding a putative fimbrial subunit protein (COG3539 domain) at its 3′-end. The amino acid sequence of this fimbrial subunit protein shares strong identity (169/367 or 46% identical residues) and similarity (226/367 or 62% similar residues) with the amino acid sequence of the adjacent (conserved) subunit-encoding gene.

### Distribution of CU fimbriae among *E. coli* pathotypes

**Figure 4:**
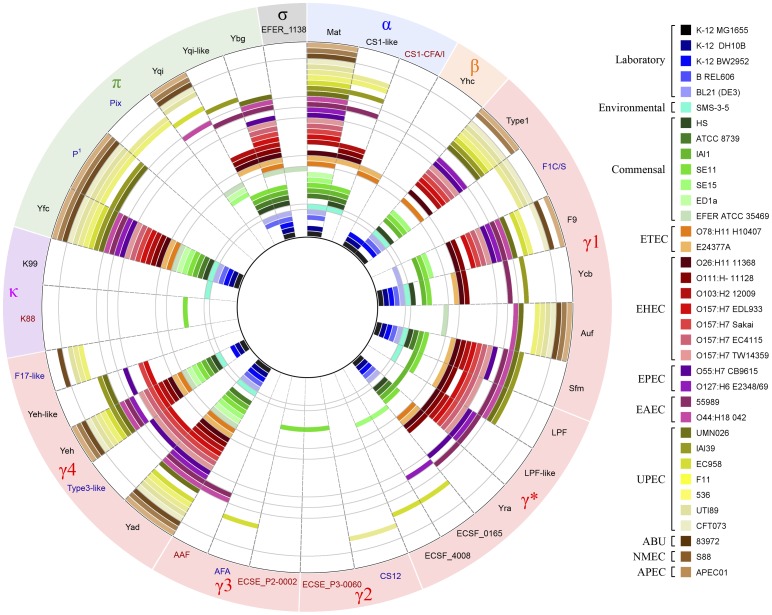
Distribution of CU fimbrial gene clusters among *E. coli* pathotypes. The inner ring represents the concatenated nucleotide sequences of the 38 fimbrial operons. Each segment is labelled in the outer ring according to the name and clade [3] of the corresponding fimbrial usher type with the intervening 36 rings displaying the presence of intact CU fimbrial gene clusters in each of the strains analysed. The legend on the right lists the colour of each strain that we included in our study, grouped according to pathogenicity class. Circular comparison was generated using BLAST ring image generator (BRIG) [69]. ^1^CFT073 contains two copies of the P fimbriae operon.

In total, 38 distinct CU fimbrial operons were identified. The distribution of each intact CU fimbrial operon was assessed with respect to *E. coli* pathogenicity class (Figure 4). Five fimbrial types were common to most pathotypes: type 1, Yad, Yeh, Yfc and Mat (Ecp) fimbriae. Type 1 fimbriae, as discussed above, represent the most well characterised CU fimbriae and mediate binding to α-D-mannosylated receptors. The *yad*, *yeh* and *yfc* CU fimbrial genes encode for functional but cryptic surface organelles, and thus their precise role in colonisation remains to be determined [35]. Recently, Yad fimbriae were shown to be associated with adherence to UM-UC-3 bladder epithelial cells and biofilm formation [53], although their expression in wild-type strains remains to be demonstrated. Mat (meningitis associated and temperature regulated) fimbriae were first identified in neonatal meningitis *E. coli* (NMEC) [54] and have subsequently also been named ECP (*E. coli* common pilus) due to their apparent ubiquitous association with most *E. coli* strains [55]. Mat (ECP) fimbriae mediate biofilm formation and adherence to cultured epithelial cells [55], [56].

Some of the CU fimbrial genes displayed a clear pathotype association. For example P, F1C/S, F17-like and Pix fimbriae genes were only found in ExPEC strains. P and F1C/S fimbriae are associated with colonisation of the urinary tract. F1C fimbriae bind to galatosylceramide targets present on epithelial cells in the kidneys, ureters and bladder as well as to globotriaosylceramide present only in the kidneys [28], [57]. S fimbriae recognize α-sialyl-2,3-galactose receptors present on the surface of host glycoproteins [58]. Pix fimbriae, although functionally characterised from an *E. coli* strain isolated from the urinary tract, do not bind to receptor targets recognized by other UPEC fimbriae [59]. The function of F17-like fimbriae has not been characterised.

Other examples of pathotype association were also apparent. CS1-CFA/I fimbriae, which contribute to intestinal colonisation [60], were strongly associated with ETEC. The Lpf and Lpf-like fimbrial types were predominantly associated with diarrheagenic *E. coli* strains, although there were some exceptions for Lpf fimbriae as they were also detected in the UPEC strains UMN026 and IAI39. Similarly, Ybg fimbriae were predominantly found in commensal and diarrheagenic *E. coli* strains (except for UPEC strain UMN026) and type 3-like fimbriae were only found in EHEC and EPEC strains. In this dataset, AAF fimbriae were only present in EAEC. AFA fimbriae, which contribute to the virulence of EIEC and UPEC [61], were only present in UPEC strain EC958 in this dataset. The Yhc, ECSE_P2-0002, ECSE_P3-0060, CS12, ECSF-4008, ECSF-0165, K88, K99 and EFER_1138 fimbriae were highly under-represented in the strains selected for our analysis.

### Distribution of fimbriae among *Escherichia* lineages

**Figure 5:**
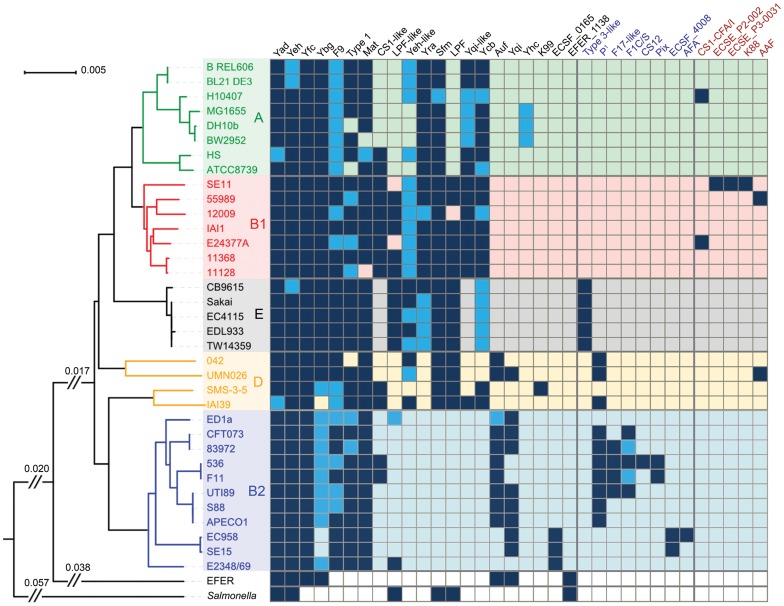
Distribution of *E. coli* fimbrial gene clusters in an evolutionary context. Left: The phylogeny of the *Escherichia* strains is displayed as inferred using the Neighbour Joining method on the concatenated nucleotide sequence of 7 housekeeping genes (∼9 kb). *E. coli* strains are colour-coded according to phylogroup (A, B1, B2, D and E). To determine CU fimbrial gene cluster ancestry, the *Salmonella* pan-genome was investigated for the presence of *Escherichia* fimbrial types. The scale indicates the number of substitutions per nucleotide. Right: The names of fimbrial types are displayed along the top of a fimbrial gene cluster matrix, with the names of PAI or plasmid-born CU fimbrial gene clusters highlighted in blue and red, respectively. Dark blue and light blue cells represent intact and disrupted CU fimbrial gene clusters, respectively. The heterogenous distribution of CU fimbrial types identified in our dataset suggests substantial acquisition and loss of CU fimbrial gene clusters during the evolution of the *Escherichia* genus. Depending on their distribution, CU fimbrial types can be classified as core-associated, clade-specific, or sporadic. ^1^CFT073 possesses two copies of the P fimbriae operon.

To examine the evolutionary history of the 35 *E. coli* and one *E. fergusonnii* strains in our dataset, we constructed a phylogenetic tree based on multi-locus sequence typing (MLST) of the concatenated nucleotide sequences of seven housekeeping genes [49]. Integration of the *Escherichia* phylogeny with the distribution of fimbrial gene clusters enabled us to evaluate the evolutionary history of CU fimbriae in the genus (Figure 5). As the majority of chromosome-borne fimbrial types occupy a single locus position, the most parsimonious evolutionary scenario suggests that the corresponding fimbrial gene clusters were acquired by a common ancestor through horizontal gene transfer or homologous recombination, and subsequently lost or disseminated vertically in its descendants. Exceptions are CU fimbriae located on PAIs. These elements are inherently prone to recombination events and can be found in a number of integration “hot-spots” (typically tRNA sites) relative to the *E. coli* chromosome backbone (Figure 2) [62]. Parsimony inference of the heterogeneous fimbrial presence (complete/partial) or absence pattern reveals extensive gain or loss of CU fimbrial gene clusters during the evolution of *E. coli* [63].

Based on their distribution in *E. coli*, we can divide the CU fimbrial types into three groups: core-associated, clade-specific, and sporadic fimbriae. The core-associated Type 1, Yad, Yeh, Yfc and Mat (Ecp) fimbriae were conserved in the vast majority of *E. coli* strains, suggesting their presence in an *E. coli* common ancestor. These genes were present as intact or disrupted operons at the same genetic locus in almost all strains examined, with only the *yfc* cluster intact in all genomes. The *E. coli mat* (*ecp*) fimbrial genes are also highly conserved in *Klebsiella pneumoniae* genomes but do not share the same syntenic location. The F9 and Ybg fimbrial gene clusters could also be considered as part of the core-associated group, however these loci are not intact in many strains.

*E. coli* population genetics have identified five major monophyletic clades (phylogroups A, B1, B2, D and E) [49]. Although these phylogroups do not correlate directly with virulence, some inferences can be made; for example ExPEC strains mainly belong to phylogroups B2 and D, whereas EHEC strains are associated with phylogroups B1 and E. The number of CU fimbrial gene clusters identified from strains in each phylogroup varied as follows: A (n = 8 strains), average of 12 (total) and 9 (intact) CU fimbriae per strain; B1 (n = 7 strains), average of 15 (total) and 13 (intact) CU fimbriae per strain; B2 (n = 11 strains), average of 11 (total) and 9 (intact) CU fimbriae per strain; D (n = 4 strains), average of 13 (total) and 11 (intact) CU fimbriae per strain; E (n = 5 strains), average of 14 (total) and 12 (intact) CU fimbriae per strain. Clade-specific fimbriae were associated with one or more *E. coli* phylogroups. An example can be observed in the case of Yqi/Yqi-like fimbriae and Auf/Ycb fimbriae. These fimbrial types occupy various locus positions on the bacterial genome and are closely related but mutually exclusive. The Auf and Yqi operons were common to the B2 phylogroup, while the Ycb and Yqi-like operons were associated with the A, B1, D and E phylogroups. The CU fimbrial profile of *E. fergusonii* is most similar to the *E. coli* B2 phylogroup, which exhibits the most ancient divergence from the A, B1, D and E phylogroups [64].

Sporadic fimbriae located on the chromosome (e.g. Yhc, K99, ECSF_0165) may represent remnants of ancient CU fimbrial gene clusters lost in the majority of strains, or, as in the case of PAI-associated fimbriae, genes that were acquired more recently. Further analysis of these fimbrial gene clusters in a larger genome dataset is required before additional conclusions can be drawn on their prevalence in the *E. coli* pan-genome. This group of fimbriae also includes plasmid-borne gene clusters, which by definition are more likely to be associated with horizontal gene transfer.

### Comparative analysis of *Escherichia* and *Salmonella* CU fimbrial gene clusters

To gain a broader insight into the evolution of CU fimbriae in *Escherichia*, the *Salmonella* pan-genome (NCBI database) was investigated for the presence of the 38 fimbrial types identified in our study. *Salmonella* and *Escherichia* diverged from a common ancestor approximately 100 million years ago [65]. Nevertheless, we identified six CU fimbrial types which were conserved in both genera; Yad, Yeh, Sfm, Lpf, Lpf-like and EFER_1138 (corresponding to Sta, Stc, Fim, Stg, Lpf and SARI_01025 in *Salmonella*, respectively) [50], [66], [67]. These CU fimbrial gene clusters clade together according to usher phylogeny and occupy an identical locus position relative to the MG1655 genome (data not shown), indicating that they are ancient and were present in the common ancestor of the two genera (Figure 5). Yeh fimbriae also occupy the same locus in *Citrobacter* [68]. Although the Yad and Yeh fimbriae are highly conserved in extant *E. coli* strains (intact in 94% and 92% of our strain database, respectively), other ancestral CU fimbrial gene clusters have been lost in one or several of the *E. coli* phylogroups. For example, Sfm fimbriae (annotated as type 1 fimbriae in *Salmonella*) were present in all *E. coli* phylogroups except for B2 and *E. fergusonii*, suggesting that the *sfm* gene cluster was acquired by an ancient ancestor of these genera and later separately lost by the *E. coli* B2 and *E. fergusonii* phylogroup progenitors. Phylogenetic analysis of the Sfm usher amino acid sequences of *Escherichia, Salmonella, Citrobacte*r and *Enterobacter* supports this hypothesis (data not shown). EFER_1138 is conserved in *E. fergusonnii*, *Salmonella*, *Citrobacter*, *Enterobacter* and *Cronobacter*, however no remnants of this archaic fimbrial gene cluster were detected in *E. coli*.

## Conclusions

CU fimbriae are cell surface-located organelles produced by many Gram-negative bacteria. These fimbriae have been best studied in *E. coli*, where they contribute to adherence, colonisation, tissue tropism and biofilm formation. The generic CU fimbrial gene cluster comprises at least four genes, encoding a chaperone, usher, major subunit and adhesin. In this study, we identified 38 CU fimbrial types from a comprehensive genome and plasmid dataset that represents a diverse array of *E. coli* strains. The majority of these fimbrial types belonged to the γ clade based on usher phylogeny, however representatives from all other previously defined clades were identified. Most of the CU fimbrial gene clusters were located in syntenic locus positions on the different *E. coli* chromosomes, and these were mapped to various locations relative to the *E. coli* K12 MG1655 reference genome. Less common CU fimbrial gene clusters were often associated with PAIs or plasmid-borne. A group of core-associated *E. coli* CU fimbriae were defined, yet interestingly few of these fimbriae have been properly characterised. The diversity of CU fimbrial gene clusters identified in this study highlights several deficiencies in our knowledge of these structural organelles. While some CU fimbriae such as type 1 and P fimbriae have been comprehensively studied, little is known about the regulation and function of many other CU fimbrial types, some of which are cryptic in nature. This study provides a framework for the effective characterisation and functional analysis of the complete subset of *E. coli* CU fimbriae, and should enable comprehensive typing of these fimbriae based on their chromosome location and evolutionary history.

## Supporting Information

Table S1 **Plasmids analysed in this study.**
(DOCX)

Table S2 **Fimbriae identified in** ***Escherichia*** **genomes and plasmids.**
(XLSX)
