# Supplementary material for: Chaperone-Usher Fimbriae of Escherichia coli
Source: PLoS One. 2013 Jan 30;8(1):e52835. doi: 10.1371/journal.pone.0052835 (PMC3559732; doi:10.1371/journal.pone.0052835)
Supplement: Table S1 — Plasmids analysed in this study. (DOCX) [file pone.0052835.s001.docx]

**Table S1:** Plasmids Analysed in this Study

| **Plasmid** | **Accession** | **Plasmid** | **Accession** | **Plasmid** | **Accession** |
| --- | --- | --- | --- | --- | --- |
| N3 | NC_015599.1 | pAPEC-O1-R | NC_009838.1 | pO55 | NC_013942.1 |
| p1ESCUM | NC_011749.1 | pAPEC-O1-ColBM | NC_009837.1 | pVM01 | NC_010409.1 |
| pO157 | NC_002128.1 | pETEC_5 | NC_009791.1 | **pSFO157** | NC_009602.1 |
| pECTm80 | NC_015472.1 | pETEC_74 | NC_009790.1 | pMG828-5 | NC_008490.1 |
| pETN48 | NC_014615.1 | pETEC_6 | NC_009789.1 | **pCoo** | NC_007635.1 |
| pO26-L | NC_011812.1 | pETEC_73 | NC_009788.1 | pColK-K235 | NC_006881.1 |
| plasmidp557 | NC_014233.1 | pETEC_35 | NC_009787.1 | p9123 | NC_005324.1 |
| **pVir68** | NC_012944.1 | pETEC_80 | NC_009786.1 | pKL1 | NC_002145.1 |
| pAPEC-O103-ColBM | NC_011964.1 | pUTI89 | NC_007941.1 | pPM18 | NC_013652.1 |
| pECOS88 | NC_011747.1 | pO157 | NC_007414.1 | pO26-CRL | NC_013728.1 |
| p2ESCUM | NC_011739.1 | pOSAK1 | NC_002127.1 | **pMAS2027** | NC_013503.1 |
| p3521 | NC_014843.1 | pO26_3 | NC_013363.1 | pColE1-H22 | NC_013589.1 |
| p53638_75 | NC_010720.1 | pO26_4 | NC_014543.1 | pEntH10407 | NC_013507.1 |
| **p53638_226** | NC_010719.1 | pCT | NC_014477.1 | pO111_2 | NC_013370.1 |
| pB171 | NC_002142.1 | pEC_L46 | NC_014385.1 | pO111_5 | NC_013368.1 |
| p75 | NC_014235.1 | pEC_L8 | NC_014384.1 | pO111_4 | NC_013367.1 |
| **p746** | NC_014234.1 | pEC_Bactec | NC_014383.1 | pRK2 | NC_005970.1 |
| p1081 | NC_014232.1 | pEC_B24 | NC_014382.1 | pLMO226 | NC_010064.1 |
| pEC14-9 | NC_013782.1 | IncQ-typepQ7 | NC_014356.1 | pVI678 | NC_008597.1 |
| pEC14_114 | NC_013175.1 | pKC394 | NC_014231.1 | pMG828-4 | NC_008489.1 |
| pColE8 | NC_012882.1 | pECOED | NC_011754.1 | pMG828-3 | NC_008488.1 |
| pAR060302 | NC_012692.1 | pIP1206 | NC_010558.1 | pMG828-2 | NC_008487.1 |
| peH4H | NC_012690.1 | pEK499 | NC_013122.1 | pMG828-1 | NC_008486.1 |
| p5217 | NC_011799.1 | NR1 | NC_009133.1 | p9705 | NC_008444.1 |
| p6148 | NC_011795.1 | pLEW517 | NC_009132.1 | pSP70 | NC_010731.1 |
| pEC278 | NC_011418.1 | pMUR050 | NC_007682.3 | pIGJC156 | NC_009781.1 |
| **pOLA52** | NC_010378.1 | pAPEC-O2-R | NC_006671.1 | pAK51 | NC_009716.1 |
| pO157 | NC_013010.1 | p1658/97 | NC_004998.1 | pIGAL1 | NC_005248.1 |
| pAPEC-1 | NC_011980.1 | R721 | NC_002525.1 | pIGRW12 | NC_010898.1 |
| pEFER | NC_011743.1 | pLEW517 | NC_009131.1 | pIGMS5 | NC_010883.1 |
| pMAR2 | NC_011603.1 | pO26_1 | NC_013369.1 | MccC7-H22 | NC_010257.1 |
| pE2348-2 | NC_011602.1 | pO111_3 | NC_013366.1 | pO113 | NC_007365.1 |
| pSE11-1 | NC_011419.1 | pO111_1 | NC_013365.1 | pFL129 | NC_005923.1 |
| **pSE11-3** | NC_011416.1 | pO26_2 | NC_013362.1 | pLG13 | NC_005019.1 |
| **pSE11-2** | NC_011413.1 | pO103 | NC_013354.1 | pIS2 | NC_004429.1 |
| **pSE11-6** | NC_011411.1 | **p55989** | NC_011752.1 | pECO29 | NC_001537.1 |
| pSE11-5 | NC_011408.1 | pEK516 | NC_013121.1 | pCol-let | NC_002487.1 |
| pSE11-4 | NC_011407.1 | pEK204 | NC_013120.1 | ColE9-J | NC_011977.1 |
| pEC4115 | NC_011351.1 | pRAx | NC_012886.1 | CloDF13 | NC_002119.1 |
| pO157 | NC_011350.1 | pO26-Vir | NC_012487.1 | pO26-S1 | NC_011266.1 |
| pSMS35_130 | NC_010488.1 | pMAR7 | NC_010862.1 | pO26-S4 | NC_011228.1 |
| pSMS35_3 | NC_010487.1 | **pO86A1** | NC_008460.1 | pO26-S3 | NC_011227.1 |
| pSMS35_4 | NC_010486.1 | pAPEC-O2-ColV | NC_007675.1 | pColG | NC_010904.1 |
| pSMS35_8 | NC_010485.1 | pC15-1a | NC_005327.1 | pIGWZ12 | NC_010885.1 |

Plasmid identifiers with corresponding accession numbers. CU operons were identified in plasmids in bold.
